# C-reactive protein as a prognostic marker in chronic obstructive pulmonary disease

**DOI:** 10.3892/etm.2013.1441

**Published:** 2013-12-09

**Authors:** ZAI-CHUN DENG, PENG ZHAO, CHAO CAO, SHI-FANG SUN, FENG ZHAO, CHAO-YUE LU, HONG-YING MA

**Affiliations:** 1 Department of Respiratory Medicine, Affiliated Hospital, School of Medicine, Ningbo University, Ningbo, Zhejiang 315020, P.R. China; 2 Department of Internal Medicine, Haining People’s Hospital, Haining, Zhejiang 314400, P.R. China

**Keywords:** C-reactive protein, chronic obstructive pulmonary disease, survival

## Abstract

The present study aimed to evaluate whether circulating C-reactive protein (CRP) levels are a biomarker of systemic inflammation and a significant predictor of future chronic obstructive pulmonary disease (COPD) outcome. During the study, 116 patients with stable COPD and 35 age- and gender-matched healthy subjects with normal pulmonary function were observed. Patient follow-up was also performed to evaluate the strength of the associations between CRP levels and future outcomes. The observations from the present study showed that serum CRP levels were significantly higher in stable COPD patients than in control subjects (4.48±0.83 vs. 1.01±0.27 mg/l, respectively; P<0.05). In addition, it was identified that a serum CRP concentration of >3 mg/l is a poor prognostic variable of COPD compared with a CRP concentration of ≤3 mg/l [hazard ratio (HR), 2.71; 95% confidence interval (CI), 1.05–6.99; P<0.05]. A quantitative synthesis of four studies including 1,750 COPD patients was performed and statistically similar results were obtained (HR, 1.54; 95% CI, 1.14–2.07; P<0.01). The present study showed that circulating CRP levels are higher in stable COPD patients and, therefore, may be used as a long-term predictor of future outcomes. These observations highlight the importance of high sensitivity CRP assays in patients with stable COPD.

## Introduction

Chronic obstructive pulmonary disease (COPD) is a major cause of chronic morbidity and mortality and is predicted to be the third leading cause of mortality worldwide by the year 2020 (1). The most significant risk factor for COPD is cigarette smoking, and for a long time COPD was considered as a consequence of local damage to small airways. However, epidemiological studies have shown that the levels of a serum marker of inflammation, C-reactive protein (CRP), are higher in patients with stable COPD than in healthy controls (2,3). In addition, CRP has been reported as a strong and independent predictor of future outcomes in individuals with COPD (4). Although the hallmark feature of COPD is airflow obstruction, it is poorly predicted by forced expiratory volume in the first second (FEV_1_) only (5,6). As CRP assays are inexpensive and convenient, CRP levels may be one of the most valuable predictors of outcomes in stable COPD patients. However, currently, CRP levels are usually only determined if an exacerbation of COPD is suspected. The patients included in other specific studies were in the acute exacerbations phase of COPD (7–9). In addition, the association between CRP and mortality in COPD patients remains conflicting rather than conclusive (10–13). It was on this background that the present study was conducted, which aimed to examine whether CRP levels in patients with stable COPD are a significant predictor of prognosis following adjustment for specific prognostic factors.

## Materials and methods

### Patients

The prospective cohort study included a total of 116 patients that had been diagnosed with COPD ≥6 months previously and had been under treatment for ≥6 months. A diagnosis of COPD was based on medical history, current symptoms and available pulmonary function tests following Global Initiative for Chronic Obstructive Lung Disease guidelines (14). To exclude patients with asthma, subjects with a history of allergic rhinitis or an improvement in FEV_1_ of >12% from the predicted values following inhalation of a bronchodilator, were not included. Patients with evidence of extensive pulmonary tuberculosis, malignancy or who were suffering from psychosis were excluded from the study. All patients with COPD were clinically stable and none had a history of respiratory infection for at least a 4-week period preceding the study. Approval for this study was obtained from the Institutional Review Board for Human Studies of Affiliated Hospital, School of Medicine, Ningbo University (Ningbo, China) and informed consent was obtained from all participating subjects.

### CRP measurement

Fasting blood samples were obtained from the patients whilst at rest, prior to any other test being performed. Serum CRP levels were measured by high sensitivity immunoturbidimetry (Beckman Coulter, Inc., Miami, FL, USA). The results were given in units of mg/l and the analytical sensitivity of this analysis was 0.1 mg/l. The cutoff point for the CRP concentration was 3 mg/l, as indicated in previous studies (10,11,15).

### Follow-up

The study was conducted between August 2009 and April 2012, with a follow-up of 32 months or until patient mortality. The follow-up was carried out by telephoning the patients or their next of kin and/or checking hospital records. Critical events were recorded by the physicians in charge of the follow-ups. Subjects who were not located at follow-up and were not known to have succumbed to their illness were considered as censored at the end of the study period.

### Statistical Analysis

The continuous variables are presented as mean ± SD and the categorical variables are presented as absolute numbers and percentages. Cox regression analysis was used to examine time to COPD mortality using hazard ratios (HR) and 95% confidence intervals (CIs). Risk measures were adjusted for age, gender, FEV_1_%pred, smoking and presence of disease. Kaplan-Meier mortality curves were created to exhibit differences in mortality by selected risk factors. Quantitative synthesis of all relevant studies was performed and the methods used have been described in detail in previous studies (16,17). The HRs of time-to-event data were directly extracted from the original study or were read off survival curves to estimate the logHR and its variance, as suggested by Parmar *et al*(18). The statistical analyses were performed using SPSS, version 13.0 (SPSS, Inc., Chicago, IL, USA) and Review Manager 5.0.17 (Cochrane Library Software, Oxford, UK). Two-tailed P<0.05 was considered to indicate a statistically significant difference.

## Results

A total of 116 consecutive COPD patients (including 75 males) were recruited into the study, as well as 35 healthy subjects (including 18 males) aged over 50 years, with no evidence of COPD. The healthy subjects were randomly selected from a population sample of subjects living in the same area as the patients.

The characteristics of the COPD patients are summarized in Table I. The majority of patients were elderly with a mean age of 71 years (range, 47–91 years) and over half were male (65%). A number of the patients had comorbid illnesses, including hypertension, diabetes mellitus, chronic gastritis, cataract and coronary heart disease. The healthy controls were matched to the COPD patients with respect to age, gender, body mass index (BMI) and smoking status. No significant differences were observed in these parameters between the healthy controls and COPD patients. However, serum CRP levels were significantly higher in the stable COPD patients than in the control subjects (4.48±0.83 vs. 1.01±0.27 mg/l, respectively; P<0.05).

**Figure 1:**
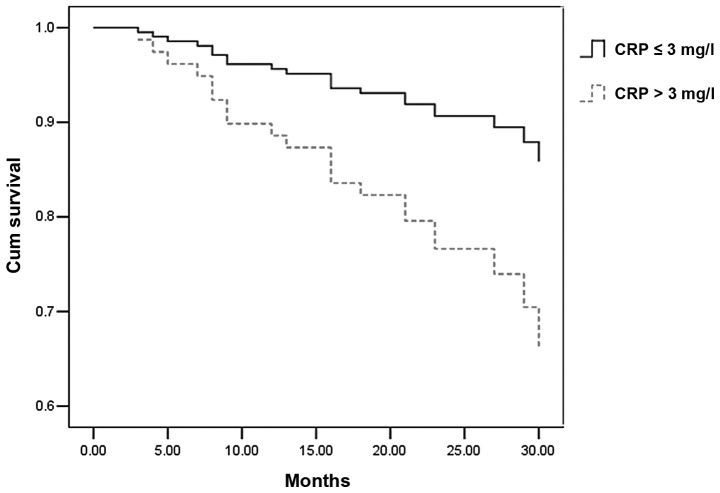
Survival curves for mortality in patients with chronic obstructive pulmonary disease according to a baseline serum CRP level of >3 mg/l or ≤3 mg/l. CRP, C-reactive protein.

At the end of follow-up, 21 patients had succumbed (18%). However, information concerning the cause of mortality of four patients was not available since contact details had been changed or through lack of cooperation from the patients’ families. When CRP ≤3 mg/l was used as the reference category, values >3 mg/l were associated with increased mortality (HR, 2.71; 95% CI, 1.05–6.99; P<0.05). Kaplan-Meier survival curves for all-cause mortality, according to CRP categories, are shown in Fig. 1. The clinical parameters between survivors and nonsurvivors were also compared. Compared with survivors, non-survivors had a high degree of airflow obstruction (FEV_1_% pred, 40.5±17.9 vs. 54.6±18.0, respectively; P<0.05) and CRP concentration (7.56±5.18 vs. 3.48±6.55 mg/l, respectively; P<0.05). However, no significant difference in age, gender and BMI was observed between the two groups.

**Figure 2:**
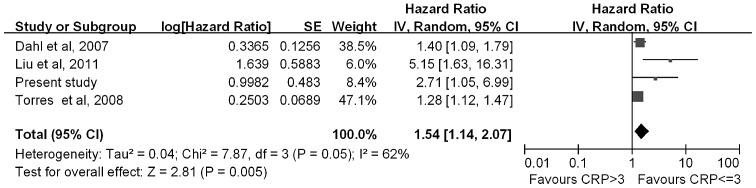
Hazard ratio of mortality with C-reactive protein level (>3 mg/l or ≤3 mg/l.) among patients with chronic obstructive pulmonary disease.

In addition, a quantitative synthesis of four studies, comprising the current study and three published studies, was performed (10,11,15). Among these studies, the median duration of the follow-ups ranged between 3 and 10 years. Risk measures were frequently adjusted for age, gender, FEV_1_%pred and smoking. As shown in Fig. 2, the HR of mortality in patients with a CRP >3 mg/l was 1.54 (95% CI, 1.14–2.07) compared with those with CRP ≤3 mg/l.

## Discussion

The present study was performed to evaluate whether circulating CRP levels are a biomarker of systemic inflammation and a significant predictor of future COPD outcomes. In this study, serum CRP levels were found to be significantly higher in stable COPD patients than in well-matched healthy control subjects. The results obtained are consistent with previous studies, indicating the presence of systemic inflammation in patients with stable COPD (2,3).

A number of independent predictors of future COPD outcomes have been identified previously, including exercise capacity (19), biomarkers of systemic inflammation (20), BMI (21,22), smoking status (23), severity of dyspnea (24), FEV_1_(5) and PaO_2_(23). Among these, the best studied and most convenient to evaluate is serum CRP levels.

The present study showed that increased serum CRP levels are a strong predictor of COPD mortality. Liu *et al* determined that a serum CRP concentration of >3 mg/l was a poorer prognostic variable of COPD compared with a CRP concentration ≤3 mg/l (11). In the study by Dahl *et al*, the HR of mortality due to COPD was 2.2-fold higher in patients with a high CRP level than in those with a low CRP level (15). The observations in the present study are in agreement with these studies. However, de Torres *et al* reported that CRP levels are not associated with survival status (10).

Considering the inconsistent results between previous studies, a quantitative synthesis of the evidence, using rigorous methods, was performed. Meta-analysis was conducted on four studies with 1,750 subjects to evaluate the association between serum CRP levels and mortality in patients with COPD. This meta-analysis indicated that a high level of serum CRP is associated with an increased risk of mortality in COPD patients.

At present, CRP levels are only determined if an exacerbation of COPD is suspected. The results of the present study have several implications, showing that stable COPD patients had a higher level of CRP than healthy controls, indicating the presence of systemic inflammation in COPD. As high sensitivity CRP assays are inexpensive and convenient, it is important for clinicians to use CRP values in stable COPD patients. The study also found that a high level of serum CRP is associated with an increased risk of mortality in COPD patients. These results indicate that selection of serum CRP concentration as a prognostic biomarker in stable COPD patients may be useful for physicians.

However, there were limitations in this study that should be acknowledged. Firstly, although the patients were clinically stable, serum CRP concentration may fluctuate slightly over time, which may affect the validity of CRP levels as a predictor marker. Secondly, various drugs and treatments among patients appear to have an unpredictable effect on serum CRP concentration.

In conclusion, the present study confirms that circulating CRP levels are higher in stable COPD patients than in healthy individuals and are a significant long-term predictor of future COPD outcomes in individuals with airway obstruction. These observations highlight the significance of high sensitivity CRP assays in patients with stable COPD.

## Figures and Tables

**Table I:** Baseline characteristics of the study participants.

Characteristics	COPD patients n=116	Healthy controls n=35	P-value
Gender			0.16
Male	75	18	
Female	41	17	
Age, years	71.0±9.0	68.9±5.1	0.09
BMI, kg/m^2^	24.6±3.2	25.8±5.7	0.12
Smoking status			0.13
Current smoker	46	14	
Ex-smoker	34	5	
Never	36	16	
FEV_1_, % predicted	44.7±12.1	90.4±6.8	<0.001
Inhaled steroid, %	61.2		
β_2_-agonist, %	61.2		
Inhaled ipratropium, %	53.4		
Theophylline, %	36.2		
Comorbid illnesses, n			
0	36		
1	52		
2	15		
3	10		
4	3		
CRP, mg/l	4.48±0.83	1.01±0.27	0.025

Data are presented as mean ± SD, n or %, unless otherwise indicated. COPD, chronic obstructive pulmonary disease; BMI, body mass index; FEV_1_, forced expiratory volume in 1 sec; CRP, C-reactive protein.
